# The cross‐lagged relationship between father absence and child problem behaviour in the early years

**DOI:** 10.1111/cch.12236

**Published:** 2015-02-24

**Authors:** E. Flouri, M. K. Narayanan, E. Midouhas

**Affiliations:** ^1^ UCL Institute of EducationUniversity College LondonLondonUK; ^2^ The Norwegian Center for Child Behavioral DevelopmentUniversity of OsloOsloNorway

**Keywords:** child behaviour, cross‐lags, emotional and behavioural problems, father absence

## Abstract

**Background:**

Father absence has negative consequences for children's behaviour. Yet research has not examined how father absence and child behaviour may influence each other. This study models the cross‐lagged relationship between father absence (non‐residence) and child problem behaviour in the early years.

**Methods:**

We used data from the UK's Millennium Cohort Study, at children's ages 3, 5 and 7 years (Sweeps 2–4). The sample was 15 293 families in which both biological parents were co‐resident at Sweep 1, when the child was aged 9 months. Child problem behaviour was assessed using the clinical cut‐offs of the Strengths and Difficulties Questionnaire (SDQ). We also investigated gender differences in the association between father absence and problem behaviour.

**Results:**

Father absence at age 3 predicted a higher probability of the child scoring above cut‐off for total difficulties at age 5, as did father absence at age 5 for total difficulties at age 7. There were no significant effects for total difficulties on father absence. Similar father absence effects were found for individual SDQ subscales. Using these subscales, we found few child behaviour effects, mostly during the preschool years: children's severe externalizing and social (but not emotional) problems were associated with a greater probability of the father being absent in the next sweep. All cross‐lagged relationships were similar for boys and girls.

**Conclusions:**

Father absence seems to be mainly the cause rather than the outcome of child problem behaviour in young UK families, and to affect boys and girls similarly. There were some child (mostly externalizing) behaviour effects on father absence, particularly in the early years.

## Introduction

There is much research on the relationship between father absence because of divorce or parental separation and child problem behaviour, including externalizing and internalizing problems (Amato 2010; McLanahan *et al*. 2013). Most research tends to find significant adverse effects of father absence. The minority of studies that report null findings appear to suffer from methodological weaknesses (such as small sample sizes and over‐adjustment, especially for endogenous variables) that likely account for the lack of significant effects (McLanahan *et al*. 2013, for a review). Although recent studies tend to be methodologically robust (Strohschein 2005; Culpin *et al*. 2013), existing research tends to suffer from two important limitations. First, it does not usually allow for children's characteristics and parents' relationships to influence each other, despite evidence for their reciprocal association (Cui *et al*. 2007). Second, it rarely explores heterogeneity fully, despite evidence, for example, that the effects of father absence on problem behaviour may depend on the child's gender (Lundberg *et al*. 2007) and the family environment prior to divorce or separation (Strohschein 2005). The timing of parents' partnership transitions also appears to be important. For example, Lansford and colleagues (2006), following a sample of children from kindergarten through 10th grade, showed that parental divorce during elementary school was more negatively related to trajectories of children's internalizing and externalizing behaviour problems than was later divorce, whereas later divorce was more negatively related to children's school grades.

Our study, using longitudinal data and a cross‐lagged design, and testing for gender differences in the estimated paths, attempted to address these issues. Using data from the UK's Millennium Cohort Study (MCS), we examined the cross‐lagged relations between father absence and problem behaviour at ages 3, 5 and 7 years, controlling for pre‐age 3 measures (at the beginning of our study period) of resources (i.e. poverty, sibship size and parents' education), inter‐parental relationship quality (i.e. conflict) and parental mental health, as well as background characteristics (parents' marital status and child's gender and ethnicity). Low levels of family resources, including income and education, are related to parental separation/divorce and lone parenthood (Kiernan & Mensah 2009; Panico *et al*. 2010) as well as child well‐being (Bradley & Corwyn 2002). Inter‐parental relationship quality is a predictor of relationship dissolution (Fomby & Osborne 2010) and child mental health (Goldberg & Carlson 2014) via parenting and behaviour modelling. Parental depression is related to both lone parenthood status and poorer emotional and behavioural outcomes in children (Kiernan & Huerta 2008). As for our background characteristics, girls are at lower risk of behavioural problems than boys (Egger & Angold 2006). The main ethnic minority groups in the UK have similar or better mental health than white British children for common disorders, and higher rates for some less common conditions (Goodman *et al*. 2008). Children with two married parents are more likely than those with two unmarried parents to have better social and emotional outcomes (Amato 2010), and unmarried resident fathers are more likely to become non‐resident.

## Method

### Sample

We used data from the MCS (http://www.cls.ioe.ac.uk/mcs), a longitudinal survey of children born in the UK during 2000–2002. The MCS was designed to over‐represent areas with high proportions of ethnic minorities in England, areas of high child poverty, and the three smaller UK countries. Ethical approval was gained from National Health Service Multi‐Centre Ethics Committees, and parents gave informed consent before interviews. Sweeps 1–4 took place when children were around 9 months, and 3, 5, and 7 years old. The complete MCS sample consists of 19 244 families. Our analytic sample (*n* = 15 293) comprised MCS singletons and first‐born twins/triplets who lived in families where both biological parents were co‐resident at Sweep 1. The 692 families who entered MCS at Sweep 2 (Plewis 2007) were therefore not included in our sample.

### Measures

The *main variables* were *father absence* and *child problem behaviour*, both measured at Sweeps 2–4. Father absence was binary‐coded (absent/present). The biological father was ‘absent’ if he was not co‐resident with the child's mother. Child problem behaviour was measured with the Strengths and Difficulties Questionnaire (SDQ; Goodman 1997), completed by the main parent (usually the mother). The SDQ consists of 25 items describing five positive and 20 negative attributes of children allocated to five subscales of five items (ranging 0–2) measuring emotional symptoms, conduct problems, hyperactivity/inattention, peer problems and prosocial behaviour. A total difficulties score (ranging 0–40) is calculated by summing the scores on the first four subscales. We identified children whose scores were above the cut‐offs for borderline/abnormal ‘problems’ (sdqinfo.net): total difficulties (13); conduct problems (2); hyperactivity (5); emotional symptoms (3), and peer problems (2), and coded all problem behaviour variables as at or below/above cut‐off. In our analytic sample, 19, 10 and 12% of children scored above cut‐off for total difficulties at ages 3, 5 and 7, respectively. For the individual problem domains, the percentages of children scoring above cut‐off at ages 3, 5 and 7, respectively, were: 49, 20, and 19% (conduct problems); 22, 16, and 18% (hyperactivity); 8, 10, and 13% (emotional symptoms); and 24, 15, and 16% (peer problems). Cronbach's alpha across sweeps ranged, respectively, 0.77–0.82 for total difficulties, 0.54–0.66 for conduct problems, 0.71–0.79 for hyperactivity, 0.52–0.64 for emotional symptoms, and 0.47–0.57 for peer problems.

The *control variables* (see Table 2 for descriptive information) were measured at Sweep 1. Family *poverty* was measured with a summary of four binary items indexing economic and material deprivation (Malmberg & Flouri 2011): overcrowding (>1.5 people per room excluding bathroom and kitchen), not owning the home, receipt of means‐tested income support, and income poverty (below the poverty line, set for equivalized net family income at 60% of the national median household income). Father's and mother's *education* was University degree or not. Father's and mother's *psychological distress* was measured with the 9‐item Malaise Inventory (Rutter *et al*. 1970). The Malaise Inventory assesses emotional disturbance and associated physical symptoms with ‘yes’ or ‘no’ responses to questions such as ‘Do you often feel miserable or depressed?’ Cronbach's alpha was 0.73 for mothers and 0.68 for fathers. Father‐reported and mother‐reported *quality of the inter‐parental relationship* was measured with the 7‐item version of the Golombok Rust Inventory of Marital State (Rust *et al*. 1990). This measure, which includes items such as ‘I wish there was more warmth and affection between us’, was administered to all MCS respondents with a full‐time resident partner at Sweep 1. Cronbach's alpha was 0.78 for mothers and 0.72 for fathers. Child's *ethnicity* was included as a set of binary dummy variables comparing Mixed, Indian, Pakistani/Bangladeshi, Black and Other ethnicity to the White reference group. Child's *number of siblings* and *gender*, and parents' *marital status* (married or not) were also included as control variables. Of the 15 293 families, 71% were married.

### Analytic approach

To examine whether father absence predicts child problem behaviour, and vice versa, at ages 3, 5 and 7 years, we estimated cross‐lagged path models with dichotomous manifest variables in Mplus 7.0 (Muthén & Muthén 1998–2012). We used the weighted least squares (WLS) estimations with robust standard errors (SEs) and corrections for means and variances (i.e. WLSMV, the default estimator in Mplus for categorical dependent variables). Therefore, our models were probit models. Our control variables (measured at age 9 months) were included to predict father absence and child problem behaviour at age 3. We allowed for residual covariances of the within‐sweep variables at each of the three sweeps. When WLS estimators are used, missingness is allowed to be a function of the observed covariates, but not the observed outcomes. To keep cases with missinginess on our control variables in the model, we specified their variances. Therefore, our complete sample size of 15 293 was maintained in all models. To account for the clustered stratified sampling design of MCS, we used probability weights with the TYPE = COMPLEX analysis command. This command computes SEs and a chi‐square test of model fit taking into account stratification and unequal probability of selection. To examine whether results differed for boys and girls, all analyses were repeated as multiple group models in Mplus. Regression coefficient estimates for boys and girls were compared using a chi‐square difference test for nested models.

## Results

### Bias analysis and descriptives

Families with co‐resident parents at Sweep 1 – those in our analytic sample (*n* = 15 293) – differed from families with absent fathers at Sweep 1 (*n* = 3951) on some of our control variables showing some sample selection bias (results available upon request). These differences are largely reflected in Tables 1 and 2. Table 1 shows that child problem behaviour was significantly related to father absence/presence at ages 3, 5 and 7, examined using chi‐square tests.

**Table 1:** Problem behaviour by father absence status (unweighted data)

SDQ scales	Age 3			Age 5			Age 7		
	Absent father	Present father	Chi‐square	Absent father	Present father	Chi‐square	Absent father	Present father	Chi‐square
	(8.6%)	(91.4%)		(13.4%)	(86.6%)		(17%)	(83%)	
	Percentage of children scoring borderline/abnormal			Percentage of children scoring borderline/abnormal			Percentage of children scoring borderline/abnormal		
Total difficulties	29.8	17.9	79.76***	18.6	8.6	153.83***	19.9	10.1	141.91***
Conduct problems	57.9	47.9	35.30***	30.9	18.6	131.77***	29.1	16.5	162.45***
Hyperactivity	30.7	21.2	46.27***	24.3	14.1	109.80***	23.8	16.4	57.82***
Emotional symptoms	12.1	7.8	20.95***	14.1	9.1	38.85***	18.2	11.4	65.56***
Peer problems	29.1	23.3	15.68***	19.5	14.1	31.76***	23.0	15.0	72.35***

***P < 0.001. SDQ, Strengths and Difficulties Questionnaire.

Note: Borderline/abnormal scores are above SDQ scale score cut‐offs. Cut‐offs are: total difficulties (13); conduct problems (2); hyperactivity (5); emotional symptoms (3) and peer problems (2). Borderline/abnormal and father being present are both coded ‘1’. Else is ‘0’.

**Table 2:** The relationship between control and main variables at age 3: descriptives and correlations (unweighted data)

Control variables (range; percentage of missing)	Father at child's age 3			Total difficulties at age 3		
	Absent (8.6%)	Present (91.4%)	r	Borderline/abnormal (18.8%)	Not borderline/abnormal (81.2%)	r
	M (SD)			M (SD)		
Number of siblings (0–9; 0%)	0.96 (1.17)	0.95 (1.04)	−.002	0.97 (1.07)	0.91 (1.00)	.03**
Quality of inter‐parental relationship (mother‐reported) (7–35; 11.4%)	25.22 (5.55)	28.22 (4.40)	.18***	26.48 (4.82)	28.38 (4.45)	−.16***
Quality of inter‐parental relationship (father‐reported) (7–35; 24%)	25.64 (4.83)	27.89 (3.99)	.14***	26.93 (4.14)	27.94 (4.06)	−.09***
Maternal psychological distress (0–9; 3.4%)	2.03 (1.91)	1.57 (1.69)	−.08***	2.40 (2.02)	1.41 (1.55)	.23***
Paternal psychological distress (0–9; 16.9%)	1.83 (1.93)	1.32 (1.52)	−.09***	1.71 (1.77)	1.27 (1.49)	.11***
Family poverty (0–4; 0.3%)	1.18 (1.10)	0.48 (0.83)	−.22***	0.89 (1.04)	0.41 (0.77)	.22***
–	**%**		**Chi‐square**	**%**		**Chi‐square**
Mother is university‐educated (0.3%)	8.3	20.8	97.75***	9.3	23.1	209.68***
Father is university‐educated (14.0%)	8.2	21.7	88.43***	11.1	23.5	140.29***
Married (0.1%)	43.1	75.4	522.02***	62.8	73.9	109.48***
Girl (0.1%)	47.2	49.2	1.72	41.7	50.9	60.36***
Mixed (0.1%)	3.7	2.1	11.36***	2.5	2.2	0.61
Indian (0.1%)	1.2	3.0	11.33***	3.3	2.3	8.43**
Pakistani/Bangladeshi (0.1%)	4.1	7.3	14.95***	10.0	3.7	151.42***
Black (0.1%)	4.0	1.7	25.38***	1.3	1.8	3.42
Other (0.1%)	1.1	1.4	0.72	1.5	1.0	5.08*

*P < .05; **P < .01; ***P < .001. SD, standard deviation.

Note: Above borderline/abnormal cut‐off (13) and father being present are both coded ‘1’. Else is ‘0’.

As expected, our control variables were generally related to both child problem behaviour and father absence at age 3 (Table 2). The relationships of the control variables with both father absence and child problem behaviour were examined using bivariate correlation coefficients and chi‐square tests (for continuous and dichotomous control variables, respectively).

Around 20% of the analytic sample missed data on father absence and child problem behaviour. Specifically, missingness at Sweeps 2, 3 and 4 was, respectively, 18, 19 and 26% for father absence, and 24, 22 and 28% for child problem behaviour.

### Cross‐lagged effects of father absence and child problem behaviour

#### Total difficulties

**Figure 1:**
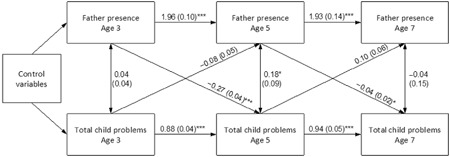
Cross‐lagged model of father absence and child total difficulties: unstandardized regression coefficients (standard errors). Control variables (measured at age 9 months) are family poverty, maternal and paternal education and psychological distress, mother‐ and father‐reported quality of the inter‐parental relationship, parents' marital status, child's number of siblings, and child's gender and ethnicity (Mixed, Indian, Pakistani/Bangladeshi, Black or ‘Other’ compared with White). Residual covariances of the within‐sweep variables are shown; effects of control variables on the age 3 variables are not shown. *P < 0.05, ***P < 0.001.

Figure 1 displays the cross‐lagged model with unstandardized regression coefficients and SEs. The model fitted the data well (Comparitive Fit Index [CFI] = 0.988; Tucker Lewis Index [TLF] = 0.981, Root Mean Square Error of Approximation [RMSEA] = 0.021). Father absence at age 3 predicted a higher probability of the child having severe problems at age 5, controlling for selection into family structure. At a smaller magnitude, father absence at age 5 predicted a higher probability of the child having severe problems at age 7, controlling for prior father absence. No significant effects were found for child problems on father absence. As with the bivariate results, most of the control variables at age 9 months were related to both father absence and child problem behaviour at age 3 years. Mother's and father's higher education, mother and father – reported higher quality of the inter‐parental relationship, married marital status, number of children in the home, and Indian or Pakistani/Bangladeshi (compared with White) ethnicity were all related to the father being present. On the other hand, family poverty and mixed or Black (compared with White) ethnic origin were related to the father being absent at age 3. Additionally, mother's and father's higher education, mother‐reported higher quality of the inter‐parental relationship, larger sibship size, having married parents and being a girl were all related to the probability of scoring at or below cut‐off for total difficulties at age 3. Family poverty, maternal and paternal psychological distress, and being Indian, Pakistani/Bangladeshi or ‘Other’ (compared with White) were related to scoring above cut‐off for total difficulties at age 3.^1^

#### Specific difficulties

We then examined the four domain subscales of the SDQ in separate models. These cross‐lagged models' unstandardized regression coefficients and SEs are shown in Table 3. As with total difficulties, father absence at age 3 predicted borderline/abnormal emotional problems at age 5, and father absence at age 5 predicted borderline/abnormal emotional problems at age 7. Also, in line with the results for total difficulties, the child's emotional problems did not predict the probability of the father being absent at any age. For both conduct and peer problems, father absence at age 3 predicted borderline/abnormal problems at age 5, and father absence at age 5 predicted borderline/abnormal problems at age 7. However, severe conduct and peer problems at age 3 also predicted the likelihood of the father being absent at age 5. Reciprocal effects were also found between ages 3 and 5 for hyperactivity. Father absence at age 3 predicted the likelihood of the child having severe hyperactivity at age 5. Furthermore, severe hyperactivity at age 3 was associated with a greater probability of the father being absent at age 5, and severe hyperactivity at age 5 was related to a greater probability of the father being absent at age 7.^2^

**Table 3:** Results (unstandardized coefficients and standard errors) of cross‐lagged models of father absence and child problem behaviour

Regression paths	Conduct problems			Hyperactivity			Emotional symptoms			Peer problems		
	B	SE	95% CI	B	SE	95% CI	B	SE	95% CI	B	SE	95% CI
Stability in father presence over time												
Age 3 > Age 5	1.97***	0.10	[1.77, 2.17]	1.98***	0.10	[1.78, 2.18]	1.96***	0.10	[1.76, 2.16]	1.96***	0.10	[1.76, 2.16]
Age 5 > Age 7	1.89***	0.13	[1.64, 2.15]	1.94***	0.14	[1.67, 2.21]	1.93***	0.14	[1.66, 2.20]	1.90***	0.13	[1.65, 2.15]
Stability in child problems over time												
Age 3 > Age 5	0.76***	0.03	[0.70, 0.82]	0.91***	0.04	[0.83, 0.99]	0.80***	0.05	[0.70, 0.90]	0.68***	0.03	[0.62, 0.74]
Age 5 > Age 7	0.74***	0.03	[0.68, 0.80]	0.83***	0.04	[0.75, 0.91]	0.71***	0.04	[0.63, 0.79]	0.79***	0.04	[0.71, 0.87]
Cross‐sectional relationships between father presence and child problems												
Age 3	0.08**	0.03	[0.02, 0.14]	0.04	0.03	[−0.02, 0.10]	0.001	0.03	[−0.06, 0.06]	0.12***	0.03	[0.06, 0.18]
Age 5	0.26***	0.07	[0.12, 0.40]	0.28***	0.08	[0.12, 0.44]	0.08	0.08	[−0.08, 0.24]	0.25***	0.07	[0.11, 0.39]
Age 7	−0.03	0.12	[−0.27, 0.21]	0.10	0.12	[−0.14, 0.34]	−0.15	0.15	[−0.44, 0.14]	0.11	0.13	[−0.15, 0.37]
F_age3_ > C_age5_	−0.27***	0.03	[−0.33, −0.21]	−0.23***	0.03	[−0.29, −0.17]	−0.10**	0.03	[−0.16, −0.04]	−0.17***	0.03	[−0.23, −0.11]
F_age5_ > C_age7_	−0.06***	0.01	[−0.08, −0.04]	−0.02	0.01	[−0.04, −0.00]	−0.04***	0.01	[−0.06, −0.02]	−0.08***	0.02	[−0.12, −0.04]
C_age3_ > F_age5_	−0.17***	0.04	[−0.25, −0.09]	−0.13**	0.05	[−0.23, −0.03]	−0.03	0.05	[−0.13, 0.07]	−0.10*	0.05	[−0.20, −0.00]
C_age5_ > F_age7_	0.06	0.05	[−0.04, 0.16]	0.15**	0.06	[−0.27, −0.03]	0.12	0.07	[−0.02, 0.26]	0.06	0.07	[−0.08, 0.20]

*P < 0.05; **P < 0.01; ***P < 0.001. CI, confidence interval; SE, standard error.

There were similarities and differences by domain in the effects of the age 9 months predictors on the age 3 outcomes. For all domains, child's sibship size and parents' higher education, higher relationship quality and married marital status were positively, and family poverty was negatively, related to the likelihood of the father being present at age 3. Compared with White, Indian and Pakistani/Bangladeshi children were more likely, and Mixed and Black children were less likely to co‐reside with their fathers at age 3. For conduct and peer problems, paternal psychological distress was associated with father absence at age 3, as well. Family poverty, maternal psychological distress and lower inter‐parental relationship quality as reported by the mother were significantly related to the child scoring above cut‐off for all difficulties. There were some distinct (by domain) patterns with ethnicity. Compared with White, Pakistani/Bangladeshi children were more likely and Black children were less likely to score above cut‐off for conduct problems. For hyperactivity, Pakistani/Bangladeshi (compared with White) children were more likely to score above cut‐off. Finally, compared with White, Indian and Pakistani/Bangladeshi children were more like to score above cut‐off for emotional symptoms, and Indian, Pakistani/Bangladeshi and mixed children were more likely to score above cut‐off for peer problems.

There were some other differences across domains, as well. Paternal psychological distress was related to child conduct problems, but no other difficulty. Mother's and father's higher education was related to a lower probability of the child having borderline/abnormal levels of hyperactivity, emotional symptoms and peer problems. Furthermore, mother's (but not father's) education was related to child's conduct problems. Having married parents was associated with a lower probability of having severe conduct and emotional problems. Larger sibship size was related to lower hyperactivity, emotional symptoms and peer problems. Lastly, girls were less likely to have borderline/abnormal levels of hyperactivity, peer problems and conduct problems, but not emotional symptoms.

### Gender differences

Our multiple‐group analysis revealed no gender differences in the cross‐lagged effects between father absence and child problem behaviour. The only significant gender difference was in the cross‐sectional relationship between father presence and conduct problems at age 3. This relationship was stronger for boys (boys: B = 0.15, SE = 0.04, *P* < 0.001; girls: B = 0.04, SE = 0.04, *P* = 0.33, chi‐square difference = 4.93, *P* = 0.03).

## Discussion

We carried out this study to investigate the cross‐lagged relationship between father absence from the home and child problem behaviour from age 3 until age 7 in a large UK child cohort. As expected, we found robust temporal stabilities of both father absence and child problem behaviour. Confirming previous findings (Panico *et al*. 2010; Pearce *et al*. 2013), we also showed strong associations between our covariates and both father absence and child problem behaviour. We also found robust effects of father absence on later problem behaviour. There was some evidence for child behaviour effects, too, although only for externalizing problems and mainly in the early years. There were no gender differences in any of the cross‐lagged relationships we modelled. Together, these findings suggest that father absence appears to be mainly the cause rather than the outcome of child problem behaviour in young UK families, and to affect boys and girls similarly. Father absence through relationship breakup may influence child behaviour through several pathways, such as via ineffective parenting, declines in household income, poor psychological functioning of the resident parent, loss of contact with the non‐resident parent, and continuing conflict and lack of cooperation among parents (Amato 2010).

Our finding that father absence in early childhood appears to increase the risk for later problem behaviour is in line with previous evidence that the effects of marital dissolution on various aspects of children's well‐being are more severe for those children whose parents separate during early childhood (Lansford *et al*. 2006). Our finding that there were few and relatively weak ‘child effects’ is in line with previous UK research that tends to find stronger parent‐to‐child than child‐to‐parent effects, particularly with regard to externalizing behaviour (Steele *et al*. 2012). Our finding of no gender differences in any cross‐lagged association is harder to interpret. The extant evidence with regard to gender differences in the association between parental divorce or father absence and childhood emotional/behavioural problems is inconclusive. It is possible, however, that the effect of father absence on problem behaviour may be different for boys and girls later in childhood or in adolescence, in line with recent UK evidence (Culpin *et al*. 2013). Following the MCS children as they grow older will test this possibility.

Our study has some important limitations. First, the cross‐lagged model that we adopted has some disadvantages despite its many strengths, including the fact that it does not explicitly consider the passage of time, and that the window of time between sweeps may be too short, or too long, to capture the reciprocal effects of father absence and child behaviour. Second, the pattern of lagged relationships we found may change as children approach adolescence. Third, our models did not account for the type(s) of family structure following the father's departure. As others have shown (Bachman *et al*. 2012), children raised apart from their biological fathers are raised in a multitude of family forms (e.g. single‐mother families, stepparent families, multigenerational families), most of which are unstable and differentially associated with child adjustment. For example, Pearce and colleagues (2013), also using the MCS data, showed that, at age 7, children in reconstituted rather than lone‐parent families had the least favourable social and behavioural outcomes, even after adjusting for family poverty. Fourth, we did not take into account father's involvement, which often appears to be a stronger predictor, than his presence in the home, of child's outcomes (Carlson 2006). Nonetheless, our supplementary analysis showed that, even after accounting for the amount of contact between non‐resident fathers and their children, children in non‐resident father families had worse outcomes than those in resident father families.

Despite these limitations, our study has important strengths. Even after taking into account reverse causality and selection into family structure, our study documented longitudinal negative effects of father non‐residence on child well‐being in this large sample of young UK families with initially co‐resident biological parents. Future research should investigate the mechanisms behind these effects.

Key messages

- Using a large sample of biological, initially two‐parent, families in the UK, we examined the cross‐lagged association between father absence (non‐residence) and child problem behaviour in early‐to‐middle childhood.
- Father absence seems to be mainly the cause rather than the outcome of child problem behaviour, and to affect boys and girls similarly.
- There were some child (mostly externalizing) behaviour effects on father absence, particularly in the early years.
- Father absence appears to carry risks for problem behaviour in early and middle childhood.

## Funding

This research was supported by a UK Economic and Social Research Council grant (ES/J001414/1) to EF, and by a personal overseas research grant to MKN from the Research Council of Norway (202438).

## Footnotes

1 To test that our results were robust to adjustment for amount of contact non‐resident biological fathers have with their children, we repeated our main analyses with a new father absence variable based on more MCS information. The original father absence variable was coded 0 = ‘absent, i.e. not living with child’ (7.1%) and 1 = ‘present, i.e. living with child’ (75.2%) with missing information for 17.7%. The new father absence variable (measured at Sweep 2) was coded 1 = ‘not living with child and no contact’ (1.2%), 2 = ‘not living with child and contact less often than a month’ (0.4%), 3 = ‘not living with child and contact at least once a month’ (0.8%), 4 = ‘not living with child and contact once or twice a week’ (2%), 5 = ‘not living with child and contact 3–4 times a week’ (1%), 6 = ‘not living with child and contact 5–6 times a week’ (0.3%), 7 = ‘not living with child and contact every day’ (0.6%), and 8 = ‘living with child’ (75.2%), with missing information for 18.6%. We found similar results (available from the authors).

2 As our data were missing at random, we tested whether WLS was the appropriate approach to dealing with missingness by repeating main analyses using Multiple Imputation (MI) for missing data. We found no differences between the two approaches (WLS and MI) in the results for the SDQ subscales. For SDQ total difficulties, a previously marginally nonsignificant regression coefficient (child total difficulties at age 3 on father absence at age 5) turned significant when the model was run using MI. However, the regression coefficient increased only slightly in actual size. As this was the sole difference between the two approaches, and a very minor one at that, we decided our original results were robust.
